# Engrailed-1 inactivation leads to scarless skin wound healing through extracellular matrix remodeling

**DOI:** 10.1016/j.gendis.2024.101484

**Published:** 2024-12-09

**Authors:** Ailing Hao, Xiangyu Dong, Yannian Gou, Aohua Li, Jiajia Li, Han Xiang, Saidur Rahaman, Yi Zhu, Hui Zhang, Wulin You, Guowei Shen, Changqi Luo, Ou Mei, Xingye Wu, Lewis L. Shi, Russell R. Reid, Tong-Chuan He, Jiaming Fan

**Affiliations:** a Ministry of Education Key Laboratory of Diagnostic Medicine, Department of Clinical Biochemistry, College of Laboratory Medicine, Chongqing Medical University, Chongqing 400016, China; b Molecular Oncology Laboratory, Department of Orthopaedic Surgery and Rehabilitation Medicine, The University of Chicago Medical Center, Chicago, IL 60637, USA; c Department of Orthopaedic Surgery, Beijing Hospital, National Center of Gerontology, Chinese Academy of Medical Sciences & Peking Union Medical College, Beijing 100005, China; d The Breast Cancer Center, Chongqing University Cancer Hospital, Chongqing 400030, China; e Department of Orthopaedic Surgery, Wuxi Hospital Affiliated to Nanjing University of Chinese Medicine, Wuxi, Jiangsu 214071, China; f Department of Orthopaedic Surgery, BenQ Medical Center, The Affiliated BenQ Hospital of Nanjing Medical University, Nanjing, Jiangsu 210019, China; g Department of Orthopaedic Surgery, Yibin Second People's Hospital, Affiliated with West China School of Medicine, Yibin, Sichuan 644000, China; h Department of Orthopedics, Jiangxi Hospital of Traditional Chinese Medicine, Jiangxi University of Traditional Chinese Medicine, Nanchang, Jiangxi 330006, China; i Department of Gastrointestinal Surgery, The First Affiliated Hospital of Chongqing, Medical University, Chongqing 400016, China; j Laboratory of Craniofacial Biology and Development, Section of Plastic and Reconstructive Surgery, Department of Surgery, The University of Chicago Medical Center, Chicago, IL 60637, USA

Hypertrophic scar and keloid are a major medical problem, which may lead to disfigurement, growth restriction, and permanent loss of function, causing severe physical, psychological, and economic burdens.^1^ When skin injury occurs, the wound heals through a dynamic series of physiological events, including blood clotting, granulation tissue formation, re-epithelialization, and extracellular matrix remodeling.^2^ However, the newly formed extracellular matrix in a scar may never achieve the flexibility or strength of the original tissue. Prior studies have suggested that the fibrotic process that occurs after skin injury may be mediated by a specific lineage of scar-prone fibroblasts in the dermis, which are responsible for scar deposition, namely engrailed-1 (EN-1) lineage-positive fibroblasts (EPFs).^3^ EN-1 is a transcription factor and plays an important role in embryonic development. In most cell types, EN-1 expression is limited to embryonic development. However, under pathological conditions, EN-1 can be re-expressed to promote phenotypic adaptation.^4^ The mechanical signaling factor YAP is associated with EPFs, establishing a link between mechanical transduction and fibrosis. Recent studies have demonstrated that EPFs play a key role in scar formation and that inhibition of YAP/EN-1 could restrict the formation of scar.^5^ However, as a downstream transcriptional factor of the YAP/TAZ pathway, EN-1's role in the pathological activation of fibroblasts and scar formation remains unclear. In this study, we investigated whether inhibition of EN-1 expression would be sufficient to suppress TGFβ1-induced fibroblast activation, extracellular matrix production, and scar formation in a skin injury model.

First, we demonstrated that the expression of EN-1 was significantly higher in mouse skin wound dermis than in normal skin (Fig. S1A). Since transforming growth factor beta 1 (TGF-β1) is a key factor in fibroblast activation in fibrotic diseases, leading to fibroblast differentiation into myofibroblasts, we used the recombinant adenovirus Ad-TGF-β to infect mouse dermal fibroblasts (mDFs) with high efficiency (Fig. S1B, panel a) and demonstrated that TGF-β1-stimulated mDFs exhibited a high level of *En-1* expression at 48 h compared with that treated with Ad-RFP as assessed by touchdown quantitative PCR (Fig. S1B, panel b).

We next constructed a recombinant adenovirus expressing *En-1*-targeted siRNAs (namely Ad-simEn1). We showed that the Ad-simEn1 effectively transduced mDFs and knocked down *En-1* expression (Fig. S1C, panels a & b). Touchdown quantitative PCR analysis showed that Ad-simEn1-mediated silencing of *En-1* expression in the Ad-TGF-β1-transduced mDFs significantly inhibited the expression of scar formation-related genes, including *Profilin1*, *Prdx1*, *Lgals1*, and *Calr* (Fig. S1D). Furthermore, we found that F-actin was significantly down-regulated after Ad-simEn1 treatment in mDFs by phalloidin staining (Fig. S1E). Through scanning electron microscopy analysis, we further observed that the reticular fibers of the extracellular matrix derived from the Ad-simEn1-transduced TGFβ1-stimulated mDFs were significantly reduced and loosely organized (Fig. 1A, panel a). Furthermore, immunofluorescence staining analysis revealed that the expression of fibronectin (FN-1), collagen type I alpha 2 chain (COL1A2), collagen type III alpha 1 chain (COL3A1), and fibrinogen (FIB) proteins was significantly down-regulated in the extracellular matrix of mDFs transduced by Ad-simEn1 (Fig. 1A, panel b), while no changes in the expression level were found in SPARC, TSP, and TNC (Fig. S1F).

**Figure 1:**
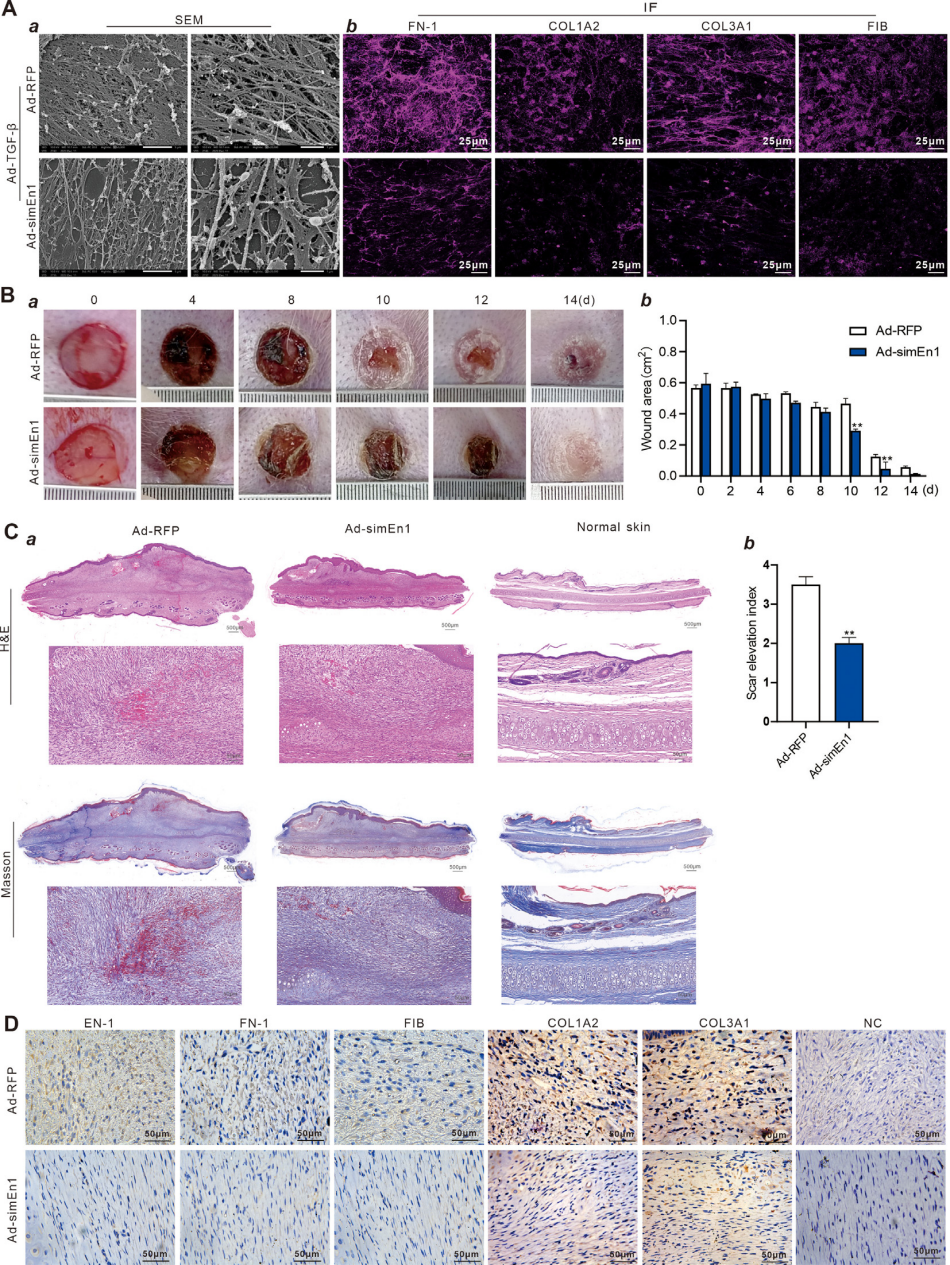
Ad-simEn1 suppresses extracellular matrix production in TGF-β1-activated mDFs *in vitro* and keloid formation in the rabbit ear skin injury model *in vivo*. **(A)** Scanning electron microscopy was used to assess the ultrastructures of decellularized extracellular matrix (a), and immunofluorescence staining was used to assess the expression of FN-1, COL1A2, COL3A1, and FIB in extracellular matrix after 7 days (b). **(B)** The adult rabbits had full-thickness skin wounds on the ventral side of the ears, and the operated rabbits were randomly divided into two groups and treated with Ad-simEn1 or Ad-RFP. The wounds were documented and measured on days 0, 4, 8, 10, 12, and 14 after treatment. ∗∗*P* < 0.01, Ad-simEn1 group versus Ad-RFP group (a & b). **(C)** Hematoxylin and eosin staining and Masson's trichrome staining of skin wound tissue after 14 days (a) and the scar elevation index was calculated from hematoxylin and eosin staining. ∗∗*P* < 0.01, Ad-simEn1 group versus Ad-RFP group (b). **(D)** Immunohistochemistry staining of EN-1, FN-1, FIB, COL1A2, and COL3A1 was used to evaluate the expression of extracellular matrix in retrieved samples from the rabbit ear skin injury model shown in Figure 1. Representative images are shown. Ad-simEn1, a recombinant adenovirus that expresses *En-1*-targeted siRNAs; Ad-RFP, a recombinant adenovirus that expresses red fluorescent protein; mDFs, mouse dermal fibroblasts; TGF-β1, transforming growth factor beta 1; FN-1, fibronectin; EN-1, engrailed-1; FIB, fibrinogen; COL1A2, collagen type I alpha 2 chain; COL3A1, collagen type III alpha 1 chain; NC, negative control.

After extensive purification of adenoviruses Ad-RFP and Ad-simEn1 (Fig. S1G), we further built a rabbit ear skin wound healing model, followed by treatment with Ad-simEn1 or Ad-RFP. Gross images of the healing wounds were taken at multiple time points, up to two weeks. The surfaces of the healing sites in the Ad-simEn1 group were significantly flatter, while apparent protruding scars were observed on the skin surfaces in the Ad-RFP group (Fig. 1B, panels a & b). Both hematoxylin and eosin staining and Masson's trichrome staining analyses revealed much fewer dermal fibroblasts and lower collagen deposition above the cartilage layer in the Ad-simEn1 group than those in the Ad-RFP control group (Fig. 1C, panel a). We also found that the scar elevation index value in the Ad-simEn1 group was almost 2 and significantly lower than that of the control group (Fig. 1C, panel b; Fig. S1H), indicating that the scar formation can be effectively inhibited by Ad-simEn1-mediated silencing of *En-1* expression at the skin wound sites. Immunohistochemistry staining analysis showed that EN-1, FN-1, FIB, COL1A2, and COL3A1 were significantly down-regulated when EN-1 functions were inhibited by Ad-simEn1-mediated silencing (Fig. 1D).

In conclusion, we blocked EN-1 with siRNAs *in vitro* and *in vivo* and discovered that knockdown of EN-1 suppressed activation of fibroblasts to promote scarless wound healing through down-regulating the expression of scar formation-related genes, especially for extracellular matrix, the ultrastructure became loose, and major protein components FN-1, FIB, COL1A2, and COL3A1 decreased. Collectively, our findings supplement the functions and mechanisms of EN-1 in the extracellular matrix of dermal fibroblasts, providing new insights for clinical approaches to prevent and reduce scar formation.

## Author contributions

**Ailing Hao:** Writing – review & editing, Writing – original draft, Visualization, Software, Methodology, Investigation, Formal analysis, Data curation, Conceptualization. **Xiangyu Dong:** Writing – review & editing, Visualization, Software, Methodology, Investigation, Formal analysis, Data curation. **Yannian Gou:** Writing – review & editing, Methodology, Investigation. **Aohua Li:** Writing – review & editing, Methodology, Investigation. **Jiajia Li:** Writing – review & editing, Validation, Resources. **Han Xiang:** Writing – review & editing, Validation, Resources. **Saidur Rahaman:** Writing – review & editing, Validation, Resources. **Yi Zhu:** Writing – review & editing, Validation, Resources. **Hui Zhang:** Writing – review & editing, Validation, Resources. **Wulin You:** Writing – review & editing, Validation, Resources. **Guowei Shen:** Writing – review & editing, Validation, Resources. **Changqi Luo:** Writing – review & editing, Validation, Resources. **Ou Mei:** Writing – review & editing, Validation, Resources. **Xingye Wu:** Writing – review & editing, Validation, Resources. **Lewis L. Shi:** Writing – review & editing, Writing – original draft, Supervision, Project administration. **Russell R. Reid:** Writing – review & editing, Writing – original draft, Supervision, Project administration, Funding acquisition. **Tong-Chuan He:** Writing – review & editing, Writing – original draft, Supervision, Project administration, Funding acquisition, Conceptualization. **Jiaming Fan:** Writing – review & editing, Writing – original draft, Visualization, Software, Methodology, Investigation, Funding acquisition, Formal analysis, Data curation, Conceptualization.

## Funding

The reported work was supported in part by research grants from the 10.13039/501100001809Natural Science Foundation of China (No. 82102696 to J.F.), the Chongqing 10.13039/501100001809Natural Science Foundation of China (No. 2024NSCQ-MSX0073 to J.F.), and the 10.13039/100000002US National Institutes of Health (No. CA226303 to T.C.H.; DE030480 to R.R.R.). T.C.H. was supported by the Mabel Green Myers Research Endowment Fund and The University of Chicago Orthopaedics Alumni Fund. Funding sources were not involved in the study design; in the collection, analysis, and interpretation of data; in the writing of the report; and in the decision to submit the paper for publication.

## Data availability

All datasets generated for this study are included in the manuscript and/or the supplementary materials. Any further inquiries about data and resource availability can be directed to the corresponding authors.

## Conflict of interests

The authors declare no conflict of interest.
