# Supplementary material for: Engrailed-1 inactivation leads to scarless skin wound healing through extracellular matrix remodeling
Source: Genes Dis. 2024 Dec 9;12(3):101484. doi: 10.1016/j.gendis.2024.101484 (PMC11804695; doi:10.1016/j.gendis.2024.101484)
Supplement: Multimedia component 1 [file mmc1.docx]

**Engrailed-1 inactivation leads to scarless skin wound healing through extracellular matrix remodeling**

**Running title: EN-1 inhibition for scar-free wound healing**

Ailing Hao^a^, Xiangyu Dong^a^, Yannian Gou^a^, Aohua Li^a^, Jiajia Li^a^, Han Xiang^a^, Saidur Rahaman^a^, Yi Zhu^b,c^, Hui Zhang^b,d^, Wulin You^b,e^, Guowei Shen^b,f^, Changqi Luo^b,g^, Ou Mei^b,h^, Xingye Wu^b,i^, Lewis L. Shi^b^, Russell R. Reid^b,j^, Tong-Chuan He^b,j^ and Jiaming Fan^a*^

^a^ Ministry of Education Key Laboratory of Diagnostic Medicine, and Department of Clinical Biochemistry, College of Laboratory Medicine, Chongqing Medical University, Chongqing, 400016, China

^b^ Molecular Oncology Laboratory, Department of Orthopaedic Surgery and Rehabilitation Medicine, The University of Chicago Medical Center, Chicago, IL 60637, USA

^c^ Department of Orthopaedic Surgery, Beijing Hospital, National Center of Gerontology, Chinese Academy of Medical Sciences & Peking Union Medical College, Beijing, 100005, China

^d^ The Breast Cancer Center, Chongqing University Cancer Hospital, Chongqing 400030, China

^e^ Department of Orthopaedic Surgery, Wuxi Hospital Affiliated to Nanjing University of Chinese Medicine, Wuxi 214071, China

^f^ Department of Orthopaedic Surgery, BenQ Medical Center, The Affiliated BenQ Hospital of Nanjing Medical University, Nanjing 210019, China

^g^ Department of Orthopaedic Surgery, Yibin Second People’s Hospital, Affiliated with West China School of Medicine, Yibin 644000, China

^h^ Department of Orthopedics, Jiangxi Hospital of Traditional Chinese Medicine, Jiangxi University of Traditional Chinese Medicine, Nanchang 330006, China

^i^ Department of Gastrointestinal Surgery, The First Affiliated Hospital of Chongqing Medical University, Chongqing 400016, China

^j^  Laboratory of Craniofacial Biology and Development, Section of Plastic and Reconstructive Surgery, Department of Surgery, The University of Chicago Medical Center, Chicago, IL, 60637, USA.

* Corresponding author

**Corresponding Author**

Jiaming Fan, MD, PhD

Ministry of Education Key Laboratory of Diagnostic Medicine &

Department of Clinical Biochemistry

College of Laboratory Medicine

Chongqing Medical University

No. 1 Medical School Road, Yuzhong District

Chongqing, 400016, China

Tel. +86 (023) 68485240

Email: [fanjiaming1988@cqmu.edu.cn](mailto:fanjiaming1988@cqmu.edu.cn)

**SUPPLEMENTAL MATERIALS**

**Materials and methods**

**Immunohistochemical (IHC) staining**

The normal and wound tissues of mice were derived from the previous research^1^ and IHC staining protocol was carried out by using the IHC staining (SP Kit, SP-9001, ZSGB-Bio, China) as described.^2^ Briefly, after antigen retrieval of the deparaffinized and rehydrated sections, the sections were subjected to immunostaining with antibodies against EN-1 (1:300 dilution; Bioss; Cat. No. bs-11744R), Fibronectin (FN-1) (1:50 dilution; Bimake; Cat. No. A5553), Fibrinogen (FIB) (1:300; Proteintech; Cat. No. 20645-1-AP), Collagen Type I (COL1A2) (1:1000; Proteintech; Cat. No. 14695-1-AP), Collagen Type III (COL3A1) (1:1000; Proteintech; Cat. No. 22734-1-AP). The proteins of interest were detected with the biotin labeled goat anti-rabbit IgG or anti-mouse IgG/streptavidin-HRP kit (SP Kit, SP-9000, ZSGB-Bio). Minus primary antibody and anti-rabbit IgG or anti-mouse IgG were used as negative controls (NC). Staining results were recorded under a bright field microscope (Leica, DM4B).

**Cell culture and chemicals**

Primary mouse dermal fibroblasts (mDFs) were obtained from the dermis of newborn C57 mouse skin as described.^1^ HEK-293 derivatives 293pTP and RAPA cells were previously described.^3^ All cells were cultured in high glucose Dulbecco’s modified Eagle’s medium (DMEM) supplemented with 10% fetal bovine serum (LONSA SCIENCE SRL, Uruguay) containing 100 units of penicillin and 100µg of streptomycin in a 5% CO_2_ incubator at 37°C.^4^ Unless indicated otherwise, all chemicals were purchased from Sigma-Aldrich (St Louis, MO, USA), Thermo Fisher Scientific (Waltham, MA, USA), or Solarbio (Beijing, China).

**Total RNA extraction and touchdown quantitative real time PCR (TqPCR) analysis**

Total RNA was isolated from the cultured cells by using the TRIZOL Reagent (Invitrogen, China), and subjected to reverse transcription with hexamer and M-MuLV reverse transcriptase (New England Biolabs, Ipswich, MA). The cDNA products were further diluted and used as PCR templates. Gene-specific PCR primers were designed by using Primer3 Plus program, and all primer sequences were shown in **Table S1**. TqPCR was carried out by using 2x SYBR Green qPCR Master Mix (Bimake, Shanghai, China) on the CFX-Connect unit (Bio-Rad Laboratories, Hercules, CA) as described.^5^ All TqPCR reactions were done in triplicate. *Gapdh* was used as a reference gene. Quantification of gene expression was carried out by using the 2^-ΔΔCq^ method as described.^6^

**Immunofluorescence (IF) staining**

IF staining was carried out as previously reported.^7^ For ECM staining, the mDFs were seeded in 24-well cell culture plates onto coverslips, treated with different conditions and the cells were removed after 7 days of cell culture, followed by being fixed with 4% paraformaldehyde at RT for 20 min and blocked with 5% bovine serum albumin (BSA) for 30 min at RT. The coverslips were incubated with the primary antibodies against FN-1 (1:50; Abcam; Cat. No. EPR23110-46), FIB (1:100; Proteintech; Cat. No. 20645-1-AP), COL1A2 (1:300; Proteintech; Cat. No. 14695-1-AP), COL3A1 (1:300; Proteintech; Cat. No. 22734-1-AP), SPARC (1:500; Proteintech; Cat. No. 15274-1-AP), TSP (1:100; Proteintech; Cat. No. 18304-1-AP), TNC (1:100; Proteintech; Cat. No. 67710-1-Ig) overnight at 4°C. After being washed, the ECM coverslips were incubated with CoraLite488-conjugated goat anti-rabbit IgG(H+L) (1:200; Proteintech; Cat. No. SA00013-2), Multi-rAb CoraLite® Plus 488-goat anti-mouse recombinant secondary antibody (H+L) (1:200; Proteintech; Cat. No. RGAM002) or CoraLite647-conjugated goat anti-rabbit IgG(H+L) (1:200; Proteintech; Cat. No. SA00014-9) for 2 h at RT. The nuclei were counterstained with DAPI (10 µg/mL). Minus primary antibody or control IgG was used as a negative control. IF results were recorded by using a laser confocal microscope (Leica TCS SP8).

**Phalloidin staining**

The mDFs were seeded in 24-well plates onto coverslips and treated with different conditions, followed by being fixed with 4% paraformaldehyde at RT for 20 min, treated with 0.4% Triton X-100 for 10 min and YF®488-Phalloidin (1:100; UElandy; Cat. No. YP0059S) for 20 min at RT. The nuclei were counterstained with DAPI (10 μg/mL). Fluorescence signals were recorded by using a laser confocal microscope (Leica TCS SP8).

**Construction and amplification of recombinant adenoviruses**

All recombinant adenoviruses used in this study, Ad-RFP, Ad-TGF-β and Ad-simEn1 were generated by using the AdEasy technology as described.^8^ The Ad-TGF-β overexpresses mouse TGF-β1 and Ad-simEn1 expresses siRNAs silencing mouse or rabbit *En-1*, both of which also express RFP and were constructed by using our recently developed methods as described.^9^ Ad-RFP was used as control. RFP signals were documented under a fluorescence microscope at 36 h after infection. For all adenoviral infections, polybrene (8 μg/mL) was added to enhance infection efficiency as reported.^10^ For direct *in vivo* injection studies, Ad-simEn1 and Ad-RFP were amplified in large scale, and purified through CsCl gradient ultracentrifugation as shown in **Fig. S1G**.

**Cell sheet/ECM culture preparation and decellularization**

The mDFs were seeded onto coverslips at 50% cell confluency in 24-well cell culture plates, co-infected with Ad-TGF-β1 and Ad-simEn1 or Ad-RFP, and cultured in complete DMEM containing 10% FBS. On the next day, 50 μg/mL of ascorbic acid was added to the medium to promote ECM secretion. The medium was changed every two days. After 7 or 10 days, the medium was removed, and the dense cell/ECM layers were washed once with PBS. Cell sheets were prepared and then permeabilized with sterile 1% Triton X-100 containing 20 mM NH_4_OH in PBS according to the procedure previously described.^11^ The ECM was washed twice with PBS, and subjected to the treatment of 10 μg/mL DNase I for 30 min at 37°C. After the decellularization process, the specimens were fixed with 4% paraformaldehyde and stored at 4 °C prior to use in IF staining and SEM analysis (see below).

**Scanning Electron Microscopy (SEM) analysis**

The mDF cell sheet and ECM samples were prepared and decellularized as described above. SEM was employed to determine the characteristics of the ECM at Chengdu Lilai Biotechnology Co., Ltd.

**Rabbit ear cutaneous hypertrophic scarring (HS) model and Ad-simEn1 treatment**

The animal experiments were approved by the Committee of Experimental Animal Administration of Chongqing Medical University, and in accordance with international ethics guidelines and the National Institutes of Health Guide concerning the Care and Use of Laboratory Animals. Four healthy New Zealand white rabbits (female, 2.0-3.0 kg) were purchased and kept in the Experimental Animal Center at the Chongqing Medical University and were randomly divided into two groups. The rabbit ear model of cutaneous hypertrophic scarring was established according to the procedure previously described.^12^ Briefly, the rabbits were anesthetized with 1% (10 g/L) pentobarbital sodium at 40 mg/kg, and six identical, 8-mm full-thickness circular cutaneous wounds were created on the ventral side of each ear using an 8-mm biopsy punch. The perichondrium in each wound bottom was completely removed from the cartilage using surgical scalpels. The wounds were exposed to air and cleaned for removal of secretions. Ad-simEn1 or Ad-RFP (10^10^ pfu, 20 μL) were added to fresh wounds. The wounds were observed and measured by using the National Institutes of Health Image J software. Any wound showing signs of infection or necrosis was excluded from the study. The animals were sacrificed at 2 weeks and the wound aeras were retrieved for histologic analysis.

**H & E staining and Masson’s Trichrome staining**

All samples for this study were fixed with 4% paraformaldehyde and subjected to paraffin embedding, followed by sectioning. Serial slides were deparaffinized, rehydrated, and subjected to H & E staining, Masson’s Trichrome staining (Masson’s Trichrome Stain Kit, G1340, Solarbio, China) as described.^13^

**Determination of scar elevation index (SEI)**

SEI was measured for histomorphometric analysis at 2x magnification from the H & E stained tissue sections. The SEI is a ratio of total wound area tissue height to the normal tissue. The height of normal tissue was determined based on the height of the unwounded dermis. An SEI of 1 indicates that the scar is of equal height to unwounded dermis; an SEI larger than 1 indicates a raised hypertrophic scarring (HS); an index of 2 indicates a wound that healed with a 100% increase in normal tissue dermal thickness. SEI was measured on multiple sections in each group using CorelDRAW 2020 software. Average SEI value for each group was calculated.

**Statistical analysis**

All experiments were performed at least three times and/or repeated in three independent batches. Data were analyzed using GraphPad Prism 8.0 and presented as mean ± standard deviation (SD). Statistical significance was confirmed by one-way analysis of variance and the student’s t test for the comparisons between groups. A value of P<0.05 was considered statistically significant.

**Table S1.** List of TqPCR primers

| **Table S1.** List of TqPCR Primers | | | |  |
| --- | --- | --- | --- | --- |
|  |  |  |  |  |
| Gene | Forward primer | Reverse primer | Accession No. |  |
|  |  |  |  |  |
| mouse *GAPDH* | ATGCCATCACTGCCACCC | GCCAGTGAGCTTCCCGTT | NM_001289726.1 |  |
| mouse *TGF-β1* | AAAGAGGTCACCCGCGTG | CTCTGCACGGGACAGCAA | NM_011577.2 |  |
| mouse *En-1* | GTCCTCTGGTCCACGCAC | GCCGCTGCTCCGTGATAT | NM_010133.3 |  |
| mouse *Cofilin1* | CCGAGAATGCACCCCTCA | GCAGGTCTGGGGCTGTTA | NM_007687.5 |  |
| mouse *Profilin1* | CTTATGGCGGACGGGACC | CTGGCCCCCAAGTGTCAG | NM_011072.4 |  |
| mouse *Prdx1* | CAGATCCCAAGCGCACCA | ATCCACAGAGCGGCCAAC | NM_011034.5 |  |
| mouse *Ltf* | CGACCTGTGGCAGCTGAA | GGGATTTTCCACCCCGCA | NM_008522.3 |  |
| mouse *Lgals1* | CTGCCTTCCCCTTCCAGC | ATGTTGAGGCGGTTGGGG | NM_008495.2 |  |
| mouse *Calr* | TGGACGGAGATGCCTGGA | TCCTTCTCCAGGTCCCCG | NM_007591.3 |  |
|  |  |  |  |  |
|  |  |  |  |  |


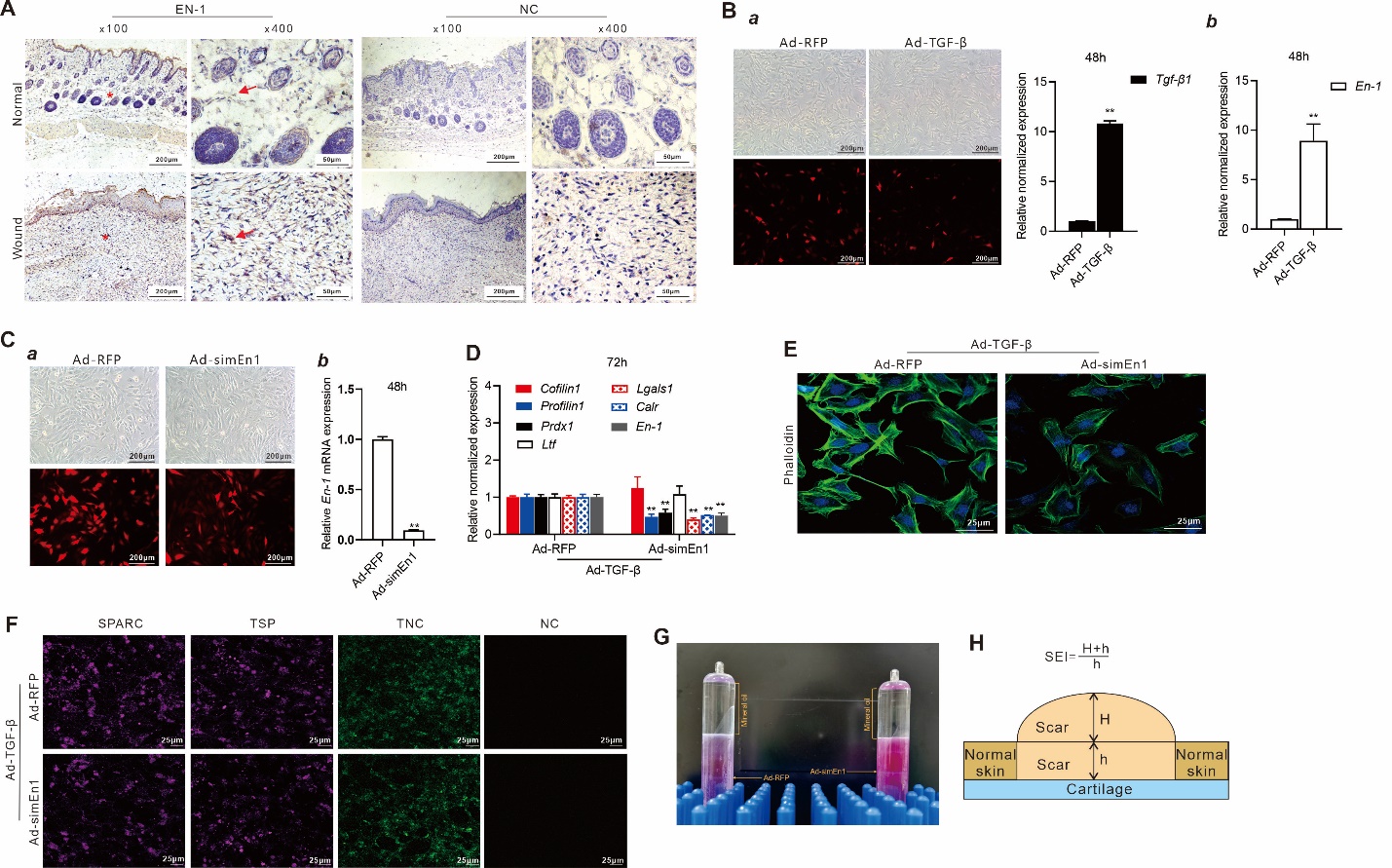


**Figure S1. Expression of EN-1 and scar formation related genes *in vivo* and *in vitro*, purification of viruses and SEI calculation formula. (A)** IHC staining of EN-1 expression at protein level in dermis in mouse wound. Positive staining in fibroblasts was indicated by red asterisks and red arrows. And IHC negative control (NC) was shown. **(B)** Subconfluent mDFs were infected with Ad-RFP or Ad-TGF-β1 respectively. The fluorescence signals were recorded at 36 h after infection and representative images are shown. Τhen total RNA was isolated for TqPCR analysis of the expression of *Tgf-β1* in mDFs at 48 h. “**” p<0.01, Ad-TGF-β1 group *vs.* Ad-RFP group **(a),** mDFs treated with Ad-RFP or Ad-TGF-β1 respectively, and Tq-PCR was used to detect the expression of *En-1* in mDFs at 48 h. “**” p<0.01, Ad-TGF-β1 group *vs.* Ad-RFP group **(b)**. **(C)** mDFs were infected with Ad-RFP or Ad-simEn1, respectively. The fluorescence signals were observed at 36 h after infection **(a)**, and total RNA was isolated for TqPCR analysis of the expression of *En-1* in mDFs at 48 h after infection **(b)**. **(D)** TqPCR analysis was carried out to detect the expression of scar formation-related genes, including *Cofilin1, Profilin1, Prdx1, Ltf, Lgals1, Calr* and *En-1* at 72 h. “**” p<0.01, Ad-simEn1 group *vs.* Ad-RFP group. **(E)** Phalloidin staining was used to assess the expression of F-actin in mDFs at 72 h. The nuclei were counterstained with DAPI. **(F)** IF staining was used to evaluate the expression of SPARC, TSP, TNC in ECM and NC was shown. **(G)** Ad-simEn1 and Ad-RFP were purified by using CsCl gradient ultracentrifugation for the *in vivo* treatment of the rabbit model of ear skin injury model. **(H)** Calculation formula and schematic diagram for the determination of scar elevation index used in the study.

**References**

1. Zhong J, Wang H, Yang K, et al. Reversibly immortalized keratinocytes (iKera) facilitate re-epithelization and skin wound healing: Potential applications in cell-based skin tissue engineering. *Bioact Mater.* 2022;9:523-540.

2. Gou Y, Huang Y, Luo W, et al. Adipose-derived mesenchymal stem cells (MSCs) are a superior cell source for bone tissue engineering. *Bioact Mater.* 2024;34:51-63.

3. Wei Q, Fan J, Liao J, et al. Engineering the Rapid Adenovirus Production and Amplification (RAPA) Cell Line to Expedite the Generation of Recombinant Adenoviruses. *Cell Physiol Biochem.* 2017;41(6):2383-2398.

4. Zhong J, Tian L, Gou Y, et al. BMP4 upregulates glycogen synthesis through the SMAD/SLC2A1 (GLUT1) signaling axis in hepatocellular carcinoma (HCC) cells. *Cancer Metab.* 2023;11(1):9.

5. Wang X, Zhao L, Wu X, et al. Development of a simplified and inexpensive RNA depletion method for plasmid DNA purification using size selection magnetic beads (SSMBs). *Genes Dis.* 2021;8(3):298-306.

6. Shu Y, Wu K, Zeng Z, et al. A Simplified System to Express Circularized Inhibitors of miRNA for Stable and Potent Suppression of miRNA Functions. *Mol Ther Nucleic Acids.* 2018;13:556-567.

7. Zhu Y, Shi Q, Peng Q, et al. A simplified 3D liver microsphere tissue culture model for hepatic cell signaling and drug-induced hepatotoxicity studies. *Int J Mol Med.* 2019;44(5):1653-1666.

8. Lee CS, Bishop ES, Zhang R, et al. Adenovirus-Mediated Gene Delivery: Potential Applications for Gene and Cell-Based Therapies in the New Era of Personalized Medicine. *Genes Dis.* 2017;4(2):43-63.

9. Ni N, Deng F, He F, et al. A one-step construction of adenovirus (OSCA) system using the Gibson DNA Assembly technology. *Mol Ther Oncolytics.* 2021;23:602-611.

10. Zhong J, Kang Q, Cao Y, et al. BMP4 augments the survival of hepatocellular carcinoma (HCC) cells under hypoxia and hypoglycemia conditions by promoting the glycolysis pathway. *Am J Cancer Res.* 2021;11(3):793-811.

11. Wen Y, Yang H, Wu J, et al. COL4A2 in the tissue-specific extracellular matrix plays important role on osteogenic differentiation of periodontal ligament stem cells. *Theranostics.* 2019;9(15):4265-4286.

12. Zhang J, Zheng Y, Lee J, et al. A pulsatile release platform based on photo-induced imine-crosslinking hydrogel promotes scarless wound healing. *Nat Commun.* 2021;12(1):1670.

13. Zhao C, Qazvini NT, Sadati M, et al. A pH-Triggered, Self-Assembled, and Bioprintable Hybrid Hydrogel Scaffold for Mesenchymal Stem Cell Based Bone Tissue Engineering. *ACS Appl Mater Interfaces.* 2019;11(9):8749-8762.
