# Supplementary material for: Engrailed-1 inactivation leads to scarless skin wound healing through extracellular matrix remodeling
Source: Genes Dis. 2024 Dec 9;12(3):101484. doi: 10.1016/j.gendis.2024.101484 (PMC11804695; doi:10.1016/j.gendis.2024.101484)
Supplement: Multimedia component 3 [file mmc3.pdf]

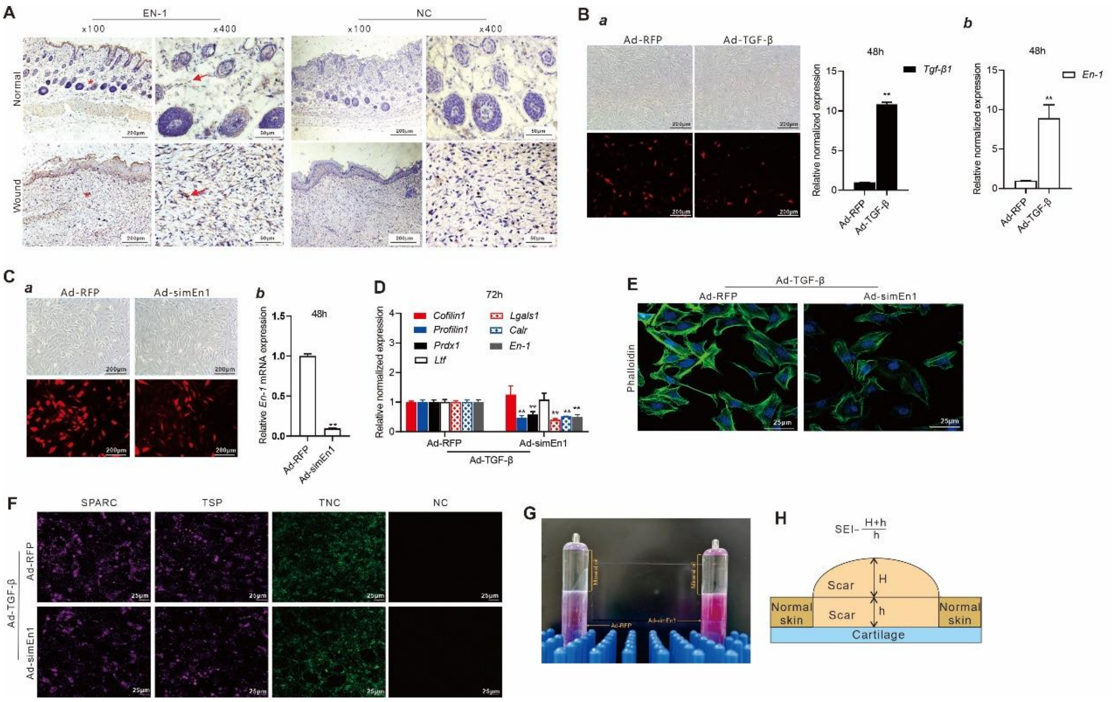

Figure S1. Expression of EN-1 and scar formation related genes *in vivo* and *in vitro*, purification of viruses and SEI calculation formula. (A) IHC staining of EN-1 expression at protein level in dermis in mouse wound. Positive staining in fibroblasts was indicated by red asterisks and red arrows. And IHC negative control (NC) was shown. **(B)** Subconfluent mDFs were infected with Ad-RFP or Ad-TGF-β1 respectively. The fluorescence signals were recorded at 36 h after infection and representative images are shown. Then total RNA was isolated for TqPCR analysis of the expression of *Tgf-β1* in mDFs at 48 h. "**" p<0.01, Ad-TGF-β1 group *vs.* Ad-RFP group **(a),** mDFs treated with Ad-RFP or Ad-TGF-β1 respectively, and Tq-PCR was used to detect the expression of *En-1* in mDFs at 48 h. "**" p<0.01, Ad-TGF-β1 group *vs.* Ad-RFP group **(b)**. **(C)** mDFs were infected with Ad-RFP or Ad-simEn1, respectively. The fluorescence signals were observed at 36 h after infection **(a)**, and total RNA was isolated for TqPCR analysis of the expression of *En-1* in mDFs at 48 h after infection **(b)**. **(D)** TqPCR analysis was carried out to detect the expression of scar formation-related genes, including *Cofilin1, Profilin1, Prdx1, Ltf, Lgals1, Calr* and *En-1* at 72 h. "**" p<0.01, Ad-simEn1 group *vs.* Ad-RFP group. **(E)** Phalloidin staining

was used to assess the expression of F-actin in mDFs at 72 h. The nuclei were counterstained with DAPI. **(F)** IF staining was used to evaluate the expression of SPARC, TSP, TNC in ECM and NC was shown. **(G)** Ad-simEn1 and Ad-RFP were purified by using CsCl gradient ultracentrifugation for the *in vivo* treatment of the rabbit model of ear skin injury model. **(H)** Calculation formula and schematic diagram for the determination of scar elevation index used in the study.
